# Construction of a CYP2J2-Template System and Its Application for Ligand Metabolism Prediction

**DOI:** 10.14252/foodsafetyfscj.D-24-00010

**Published:** 2024-12-20

**Authors:** Yasushi Yamazoe, Norie Murayama

**Affiliations:** 1 Division of Drug Metabolism and Molecular Toxicology, Graduate School of Pharmaceutical Sciences, Tohoku University, 6-3 Aramaki-Aoba, Aoba-ku, Sendai 980-8578, Japan; 2 Division of Risk Assessment, National Institute of Health Sciences, Tonomachi 3-25-26, Kawasaki-ku, Kawasaki 210-9501, Japan; 3 Showa Pharmaceutical University, Machida, Tokyo 194-8543, Japan

**Keywords:** CYP2J2-mediated metabolism, fused-grid Template, ligand immobilization, poor and good substrates, simulation of ligand-interaction on Template.

## Abstract

A Template system for the understanding of human CYP2J2-mediated reactions was constructed from the assembly of the ligands with the introduction of ideas of allowable width, Trigger-residue and the residue-initiated movement of ligands in the active site, which were in common with other Template* systems for human CYP1A1, CYP1A2, CYP2B6, CYP2C8, CYP2C9, CYP2C18, CYP2C19, CYP2E1, CYP3A4, CYP3A5, and CYP3A7 (Drug Metab Pharmacokinet 2016, 2017, 2019, 2020, 2021, 2022, 2023, 2024, and in press 2024). CYP2J2 system also includes ideas of bi-molecule binding of ligands on the Template. From their placements on the Template and rules for interaction modes, verifications of good and poor substrates, regio/stereo-selectivity, and inhibitory interaction became available faithfully for these ligands. The refined CYP2J2-Template system will thus offer reliable estimations of this human CYP catalysis toward ligands of diverse structures, together with their deciphering information to lead to judgments.

## Introduction

Cytochrome P450 (CYP) is involved in the metabolisms of hydrophobic chemicals. These enzymes belonging CYP1 to CYP4 gene families are involved in the metabolism of diverse structures of hydrophobic chemicals including medicines, endobiotics, pollutants, and other industrial chemicals.

CYP2J2 was initially characterized as an arachidonic acid epoxidase highly expressed in the human heart^1^^)^. This enzyme is unique to have high levels of extrahepatic tissue expression such as heart, small intestine, lung, kidney, brain, and pancreas^2^^,^^3^^)^.

This enzyme is now known to have distinct substrate specificity to mediate the metabolisms of terminal portions of slender-shaped substrates such as astemizole^4^^,^^5^^)^ and terfenadine^6^^)^. Of course, CYP2J2 also mediates several small-sized ligands such as *R*-5-hydroxythalidomide^7^^)^, pomalidomide^7^^)^, and benzphetamine^8^^)^.

Several three-dimensional homology models of CYP2J2 were constructed based on the crystal structure of CYP2 enzymes and the docking of ligands was examined^9^^,^^10^^,^^11^^,^^12^^)^. Considerable differences are observed in the active site volumes among the reports^9^^)^. Despite the availability of 3D homology models of CYP2J2, the metabolism prediction of ligands, particularly small-sized ones, is still far from being established.

Studies using recombinant human CYP enzyme preparations afforded considerable amounts of experimental data on ligand interactions at individual CYP enzyme levels for more than three decades. Using these advantages, we have been developing *in silico* systems to understand human CYP-mediated metabolisms by way of constructing ligand-accessible spaces through the assembly of ligands and to understand the modes of interactions of CYP residues with ligands in the active site. Our studies adopted fused-hexagonal grid systems for the Template constructions of human CYP1A1^13^^)^, CYP1A2^14^^,^^15^^,^^16^^)^, CYP2B6 (in press^17^^)^), CYP2C8^18^^)^, CYP2C9^19^^)^, CYP2C18^20^^)^, CYP2C19^21^^)^, CYP2E1^22^^)^, CYP3A4^23^^,^^24^^,^^25^^)^, CYP3A5^24^^)^, and CYP3A7^24^^)^. Both Facial- and Rear-walls are set to indicate allowable widths as Width-gauge on these Templates. Facial- and Rear-walls stand in parallel in the facial and rear sides of Templates except CYP2E1. Templates of CYP2B6, CYP2C8, CYP2C9, CYP2C18, and CYP2C19 share Shelf and left-side border (Left-end) on the left side areas. Trigger-residues migrating from their original positions push ligands to contact with Site of oxidation, and then initiate catalyses. These Template systems combined with ideas of ligand-interacting modes were established through reciprocal comparison of simulation and experimental results. Placements of ligands on Template systems of CYP2B6 (360), CYP2C8 (380), CYP2C9 (578), CYP2C18 (191), and CYP2C19 (499) offered the information on sites of metabolisms regio- and stereo-selectively with more than 99% accuracies (numbers of reactions tested are indicated in parentheses). Considering these circumstances, Shelf, Left-end, and Width-gauge were preliminarily examined as possible components of CYP2J2-Template. Simultaneous plural-contact of ligands with Rear-wall was also tested as an essential interaction. These ideas were reasonably accepted for CYP2J2 ligands in the preliminary experiments. CYP2J2-Template has thus been constructed from assembling the ligand structures in the present study.

## 2. Materials and Methods

Experimental information on the substrate specificities of CYP2J2 and metabolisms of the substrates was obtained from the literature. The published data on recombinant CYP2J2 systems were used preferably because of the direct reflections of CYP2J2 properties. Chem3D (version 5 for Mac OS, CambridgeSoft, Cambridge, MA), ChemBio3D (version 12 for Windows, CambridgeSoft), and ChemBioDraw (versions 11 and 13 for Mac OS, CambridgeSoft/PerkinElmer) were used to construct two-dimensional (2D) or 3D structures of ligands and to overlay compounds on Template.

Substrates of CYP2J2, except for polyaromatic hydrocarbons (PAHs), take various conformations due to their flexibility. Before Template application, chemical shapes are modified into their flattened form(s). The flatted or stretched shapes of 3D structures were tried to sit on Template. Conformations of the structures were then modified to fit within Template, considering the bond-energy barrier using MM2 function of Chem3D and specific interactions at distinct regions of Template. As described in the Results section, suitable ligand molecules are defined after checking several criteria such as MM2 value (<0.1), Wall contacts, specific point occupancies, and Trigger-residue contact.

Carbon, oxygen, nitrogen, sulfur, chlorine, and fluorine atoms of 3D ligand structures in figures are indicated with gray, red, blue, yellow, green, and khaki symbols, respectively. The hydrogen atoms of the substrates were not considered for the placement.

Templates consist of hexagonal grids and sticks. The sitting of substrate atoms at each corner of the hexagonal grids (termed Rings) was evaluated as occupancy. Some atoms, placed not exactly at the corner, were accepted if these atoms stayed within the Template area or at specific defined sites. The placement of ligands is expressed in a hyphen-linked form, such as Rings A-B-C, to trace the occupancy of chemical molecules on Template. The branching part is indicated in the bracket.

CYP2J2 ligands were assumed to migrate from Entrance to Site of oxidation without changing the conformation. Thus, ligands enter Template as the same conformations observed at Site of oxidation.

Chemicals including a lactone moiety are often ionized at neutral pH ranges. These lactones were treated as ionizable groups for the application of substrates in ways similar to other CYP Template systems^14^^,^^19^^,^^23^^)^. Thus, non-rigid lactone rings are not allowed into contact with Rear-wall and with Trigger-residue of CYP2J2 in general.

Several template-terms are defined to explain ligand interactions with CYP2J2 Template. These terms are listed in a separate section as “Terms used for Template system”.

## 3. Results

### 3.1 Construction of CYP2J2-Template

A total of twenty-four reactions of CYP2J2 substrates were picked up at random to reconstitute the ligand-accessible space (**Table 1**).

**Table 1.** List of ligands used for Template construction

Ligand	Reaction	Reference
ABT-107	indole oxidation	^50^
Albendazole	*S*-oxidation	^29^
	ω-oxidation	
Astemizole	*O*-demethylation	^5^
AZB6738	*N*-oxidation	^31^
Benzphetamine	*N*-demethylation	^8^
Bufuralol	1’-oxidation	^27^
Chlorzoxazone	6-oxidation	^27^
Danazol	oxazole oxidation	^32^
DB 289	*O*-demethylation	^37^
*R*-Eperisone	ω/ω -1 oxidation	^51^
17α-Ethynylestradiol	acetylene oxidation	^52^
*R*-5-Hydroxythalidomide	aromatic oxidation	^7^
Ibrutinib	epoxidation	^53^
	aromatic oxidation	
	piperidine oxidation	
Ketoconazole	methyl oxidation	^10^
Linezolid	morpholine oxidation	^26^
Luciferin-2J2/4f12	*O*-dealkylation	^46^
Magnolin	*O*-demethylation	^36^
*R*-Pomalidomide	aromatic oxidation	^7^
Rivaroxaban	morpholine oxidation	^38^
STS-135	adamantane ring oxidation	^40^
Thioridazine	2-*S*-oxidation	^28^

**Fig. 1.**
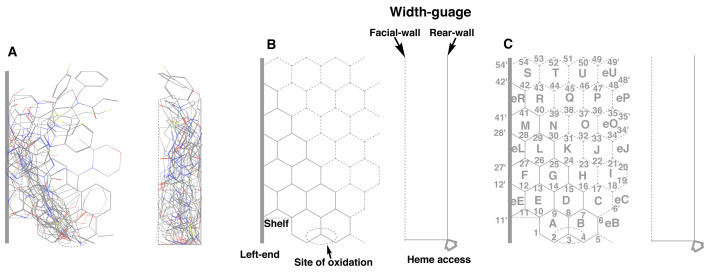
Construction of Template 3D structures of CYP2J2 substrates described in Table 1 were assembled considering that their sites of metabolism came close on a plane. The site of metabolism (Site of oxidation) is shown as a dotted oval. Their 90°-rotated structures are shown on the right side and included in a column. A vertical bar was set at the left end (A). An overlapped area was extracted as a hexagonal-grid Template. Site of oxidation is shown as a dotted oval. Two structures, termed Shelf and Left-end are located on the left side borders. The allowable width termed Width-gauge was set between Facial-wall (dotted line) and Rear-wall (solid line). A gray-colored arrow indicated the direction of heme access (B). The Ring names and Position numbers were assigned in alphabetical order (A-U) and from 1 to 54, respectively. Parts of side-end regions were also assigned as eB, eC, eE, eJ, eL, eO, eP, eR, and eU (C).

Assuming that a ligand-accessible space of CYP2J2 keeps some of the mutual structural properties of other human CYP2 enzymes such as Shelf, Left-end, and allowable width, the 3D structures of the CYP2J2 ligands were assembled on an area in ways to arrange their oxidized sites at a specific area (Fig. 1A).

Their sites of oxidation were focused at a specific place of the bottom space shown as a dotted circle (Fig. 1A). All the 3D structures were kept within Facial- and Rear-walls, which stood in parallel with 1.5 Ring-size distance (termed Width-gauge, Figs. 1A and B) after the minimization of the allowable width as much as possible.

Localizations of Shelf and Left-end were observed in the left side area (Fig. 1B). No ligands thus entered spaces under Shelf and left side of Left-end.

A core area was extracted as a fused hexagonal-grid shape from the densely overlapped part (Fig. 1B). The combined Ring construct was termed “CYP2J2-Template”. The Rings and Positions were assigned in alphabetical order (A-V plus eE, eL and eR) and numerical numbers from 1 to 54, respectively (Fig. 1C). Parts of right-side end regions were also assigned as eB, eC, eJ, eO, eP, and eU. Shelf structure was expected between Left-end and Position 10 of a junction of Rings A and E (dotted line) on Template. The heme-bound oxygen atom was assumed to access a constant position at the bottom of Template from the rear side direction.

### 3.2 Minimal Occupancy at the Bottom Region

**Fig. 2.**
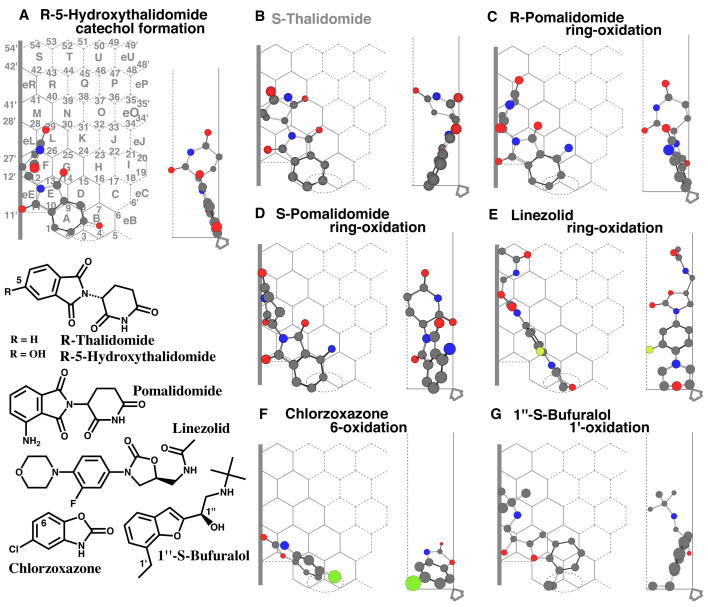
Placements of small-sized ligands Placements of *R*-5-hydroxythalidomide catechol formation (A), of ring oxidations of *S*-thalidomide (B) and *R*- (C) and *S*-pomalidomide (D), of linezolid ring-oxidation (E), of chlorzoxazone 6-oxidation (F), and 1”-*S*-bufuralol 1’-oxidations (G), are shown as cylindrical shapes of 3D structures on full-size (A) and trimmed Templates (B-G). Ligands are also indicated as 2D structures on the left bottom parts with parts of their chemical positions. Functional and non-functional placements are distinguished with dark- and gray-colored structure names, respectively.

CYP2J2 mediates the aromatic oxidation of *R*-5-hydroxythalidomide, but not of *R*-thalidomide and *S*-thalidomide^7^^)^. A placement was available for the aromatic oxidations of *R*-5-hydroxythalidomide at Rings A(B)-E(G)-eE-F-L (**Fig. 2A**). Placements for *R*-thalidomide and the *S*-isomer were also constructed at Rings A-E(G)-eE-F-L (Data not shown) and at Rings A-E(D/eE)-F(eL)-L (**Fig. 2B**), respectively. All the three placements fulfilled Simultaneous plural-contact with Rear-wall, Left-end contact, Facial-wall contact, and occupancy at Site of oxidation, but only the good substrate, *R*-5-hydroxythalidomide, had occupancy at the right side of the junction of Rings A and B on Template.

Pomalidomide also undergoes CYP2J2-mediated oxidation of the aromatic ring^7^^)^.

A placement of *R*-pomalidomide was available for the aromatic oxidation at Rings A(D)-E(G)-eE-F-L (Fig. 2C). A placement for the aromatic oxidation of *S*-pomalidomide was also generated at Rings A(D)-E(G)-eE-F-eL (Fig. 2D). The 4-amino part served Simultaneous plural-contact with Rear-wall and thus would provide a contact of the 5,6-position with heme-oxygen. Both the placement had the occupancies at the right side of the junction of Rings A and B on Template. The requirement of the right-side occupancy was further examined with small-sized CYP2J2 substrates, linezolid, chlorzoxazone, and bufuralol.

CYP2J2 mediates the oxidation of morpholine ring of linezolid^26^^)^.

A placement of linezolid was generated for the morpholine ring oxidation at Rings B-A-E-F-eL-M (Fig. 2E). The three rings contacted simultaneously with Rear-wall, and the fluorine atom of the middle ring attached with Facial-wall. The oxazoline ring and acetamide sidechain were attached to Left-end. Differing from *R*-5-hydroxythalidomide and pomalidomide, the linezolid molecule had no Shelf contact at Position 10. This molecule was, however, expected to stand at Site of oxidation due to the rigid skeleton and stable contact with Left-end. A part of the morpholine ring was located at Ring B.

Chlorzoxazone undergoes CYP2J2-mediated 6-oxidation although the rate is low^27^^)^.

A placement was available for the 6-oxidation at Rings B-A-E-eE (Fig. 2F). This molecule contacted with Shelf and Left-end, in addition to the Simultaneous plural-point contact with Rear-wall. The chlorine atom was sat at Ring B. The 6-oxidation was expected from the sitting at Site of oxidation on Template.

CYP2J2 mediates 1’-oxidation of bufuralol^27^^)^. A placement of 1”-*S*-bufuralol was generated for the 1’-oxidation at Rings A-D(B)-E-eE-F(L)-eL (Fig. 2G). The 1”-*S*-bufuralol molecule contacted with Left-end and Shelf, along with Simultaneous plural contact with Rear-wall. The benzyl part around Position 3 would be oxidized to yield the 1’-oxidized metabolite.

The results consistently suggested the essential functional role of Ring B occupancy of CYP2J2 ligands.

Catalytic reactions of CYP1A, CYP2B6, CYP2C, CYP2E1, and CYP3As occurred after the immobilization of ligands with Trigger-residues on these Template systems. These Trigger-residues migrated to fasten ligands after the arrival of ligands near the active sites. Trigger-residue of CYP2J2 was also assumed to hold ligands on the Template from the sitting of the substrates described above (Figs. 1 and 2).

Trigger-residue of CYP2J2 was expected to move to the left direction from eB region, but might not enter Ring A. If so, the thalidomide molecule would not be immobilized on CYP2J2-Template. The idea was consistent with the poor substrate specificity of thalidomide (Fig. 2B).

### 3.3. Trigger-residue and The Interaction with Ligands

CYP2J2 mediates the *N*-demethylation of benzphetamine^8^^)^.

**Fig. 3.**
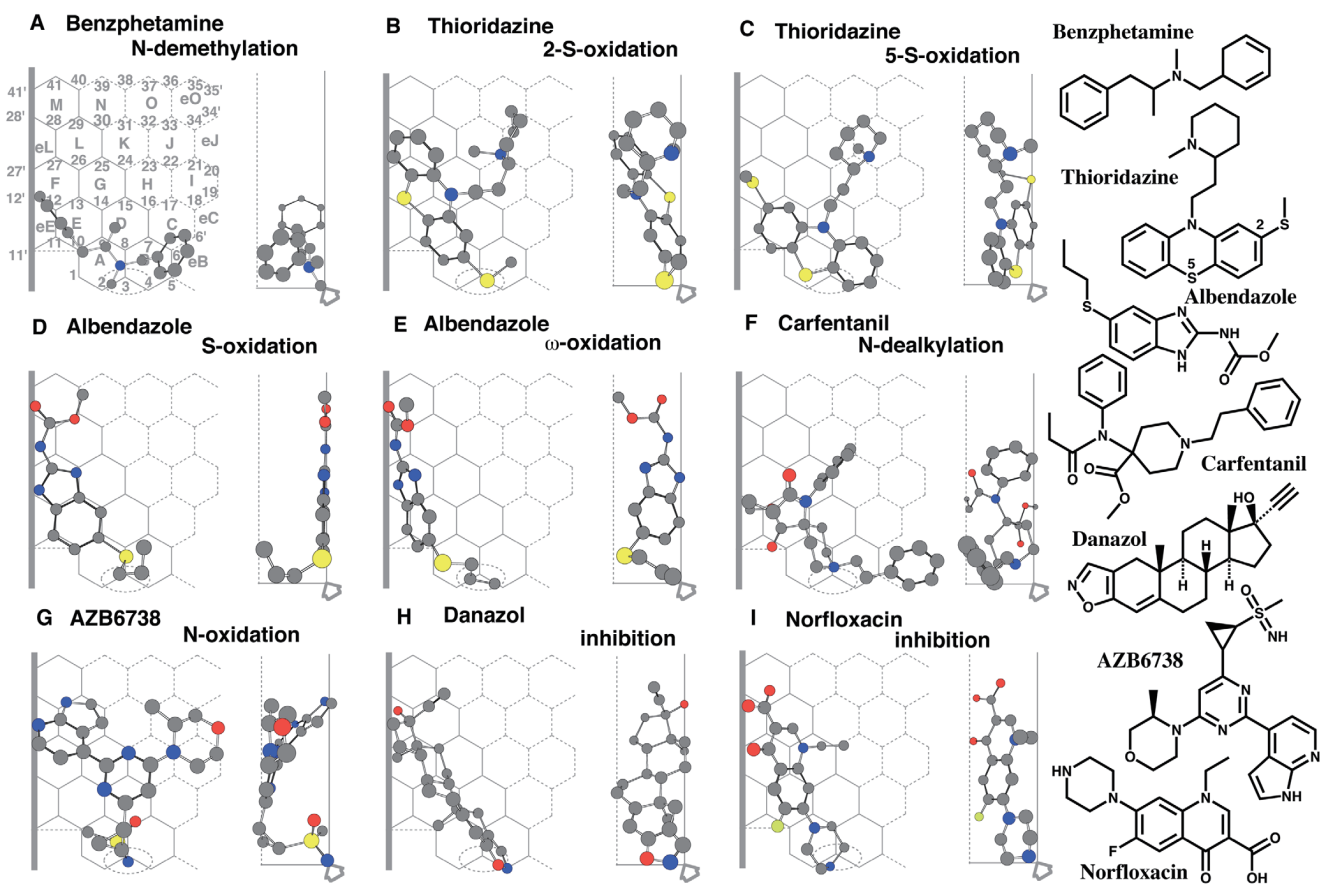
Ligand interaction with Trigger-residue Placements of benzphetamine *N*-demethylation (A), of thioridazine 2-*S*- (B) and 5-*S*-oxidations (C), of albendazole *S*- (D) and ω-oxidations (E), of carfentanil *N*-dealkylation (F), of AZB6738 *N*-oxidation (G), of danazol inhibition (H), and also of norfloxacin inhibition (I) are shown as cylindrical shapes of 3D structures on trimmed Template. Ligands are also indicated as 2D structures with parts of their chemical positions.

A placement of benzphetamine was generated for the *N*-demethylation at Rings C-B-A(D)-E-eE-F (Fig. 3A). The benzphetamine molecule satisfied Simultaneous plural-contact with Rear-wall, and also contacts with Left-end and Shelf. The *N*-benzyl part, exceeding the junction of Rings A and B, sat on the facial side at Rings B and C on Template. The *N*-methyl part was located at the rear side around Position 3 to be oxidized.

Thioridazine undergoes CYP2J2-mediated 2-*S*- and 5-*S*-oxidations^28^^)^.

Placements of thioridazine for the 2-*S*- and 5-*S*-oxidations were available at Rings B-A-E-F(G-H-K-J-O)-eL-L (Fig. 3B) and at Rings B-A(D-H-J-K)-E-F (Fig. 3C), respectively. The 2-*S*-methyl part was located at Ring B for the 2-*S*-oxidation placement in the former placement (Fig. 3B). The *S*-methyl-substituted phenyl part sat on Shelf and the 5-sulfur part was around Position 2 in the latter placement (Fig. 3C).

CYP2J2 mediates *S*- and ω-oxidations of albendazole^8^^,^^29^^)^.

Two placements of albendazole for the *S*-oxidation and ω-oxidation were generated at Rings A(B)-E-F-eL-M(L)-N (Fig. 3D) and Rings A(B)-E-F-eL-M (Fig. 3E), although distinct placements were possible to be constructed for both the reactions. Sittings around Site of oxidation of both molecules (Figs. 3D and E) were stabilized with Shelf and Left-end contacts. The interactions of albendazole molecules with Trigger-residue were expected to occur at Ring B.

Carfentanil undergoes CYP2J2-mediated *N*-dealkylation^30^^)^.

A placement of carfentanil was constructed for the *N*-dealkylation at Rings B-A-D-E(eE)-G(H)-K plus a right-side space of Ring B (Fig. 3F). The propionyl and methyl carboxylate parts hit to Left-end and the latter also contacted with Shelf. The oxidation of the piperidine nitrogen atom would result in the *N*-dealkylation of carfentanil. The terminal phenethyl part stayed at eB region of CYP2J2-Template.

CYP2J2 mediates the *N*-oxidation of AZB6738 to lead to the deamination product^31^^)^.

A placement of AZB6738 was generated for the *N*-oxidation at Rings A(B)-D(H-I-J)-G-F(eL)-L (Fig. 3G). The pyrrole and methylsulfoimidoyl parts contacted simultaneously with Rear-wall. The pyridine and methylsulfoimidoyl parts served contacts with Left-end and Shelf, respectively.

The results of benzphetamine, thioridazine, albendazole, carfentanil, and AZB6738 offered an idea of Trigger-residue migration as follows. After the descending of ligands near Site of oxidation, Trigger-residue appeared at a space right-side of Ring B and then migrated to the left direction to contact with ligands. Trigger-residue was able to migrate maximally until Ring B region, but not to enter Ring A, through the path located around Rear-wall.

The idea of Trigger-residue interaction was verified also with inhibitors of CYP2J2.

Danazol inhibits oxidations of typical CYP2J2 substrates like astemizole and albendazole^32^^)^.

A placement of danazol was available at Rings B-D-E-F-eL-L (Fig. 3H). The molecule had Simultaneous plural-contact with Rear-wall. The methyl part at the angular position (of rings A and B) sat on Shelf. The nitrogen atom of the isoxazole ring, which was located at the right-side of Rings A and B junction (Positions 3 and 8), was expected to interact with heme for the inhibition. Trigger-residue was expected to interact with the isoxazole part at Ring B.

A couple of danazol metabolites derived from the isoxazole cleavage are isolated *in vivo* in the urines of humans^33^^,^^34^^)^. These metabolite formations were possible partly due to CYP2J2-mediated reductive cleavage of the isoxazole ring^28^^)^, although no definitive data showing the CYP2J2 involvement was available at present.

Norfloxacin inhibits CYP2J2-mediated astemizole *O*-demethylation^35^^)^.

A placement of norfloxacin for the inhibition was available at Rings A(B)-E-F-G(H)-L(eL)-M (Fig. 3I). The fluorine atom sat on Shelf and the *N*-ethyl and piperazine parts contacted with Rear-wall. The terminal nitrogen atom of the piperazine ring was expected to interact with heme. Trigger-residue would interact with the piperazine part at Ring B.

The results with inhibitors were consistent with those of good substrates of CYP2J2 and supported the mode of Trigger-residue interaction on CYP2J2-Template described above.

### 3.4 Placement of Slender Shape Ligands and Left-end

CYP2J2 mediates nearly selectively the 4-*O*-demethylation of the 3,4,5-trimethoxyphenyl part of magnolin (M-2 formation) and only a trace of the demethylation of the 3,4-dimethoxyphenyl part (M-1 formation)^36^^)^.

**Fig. 4.**
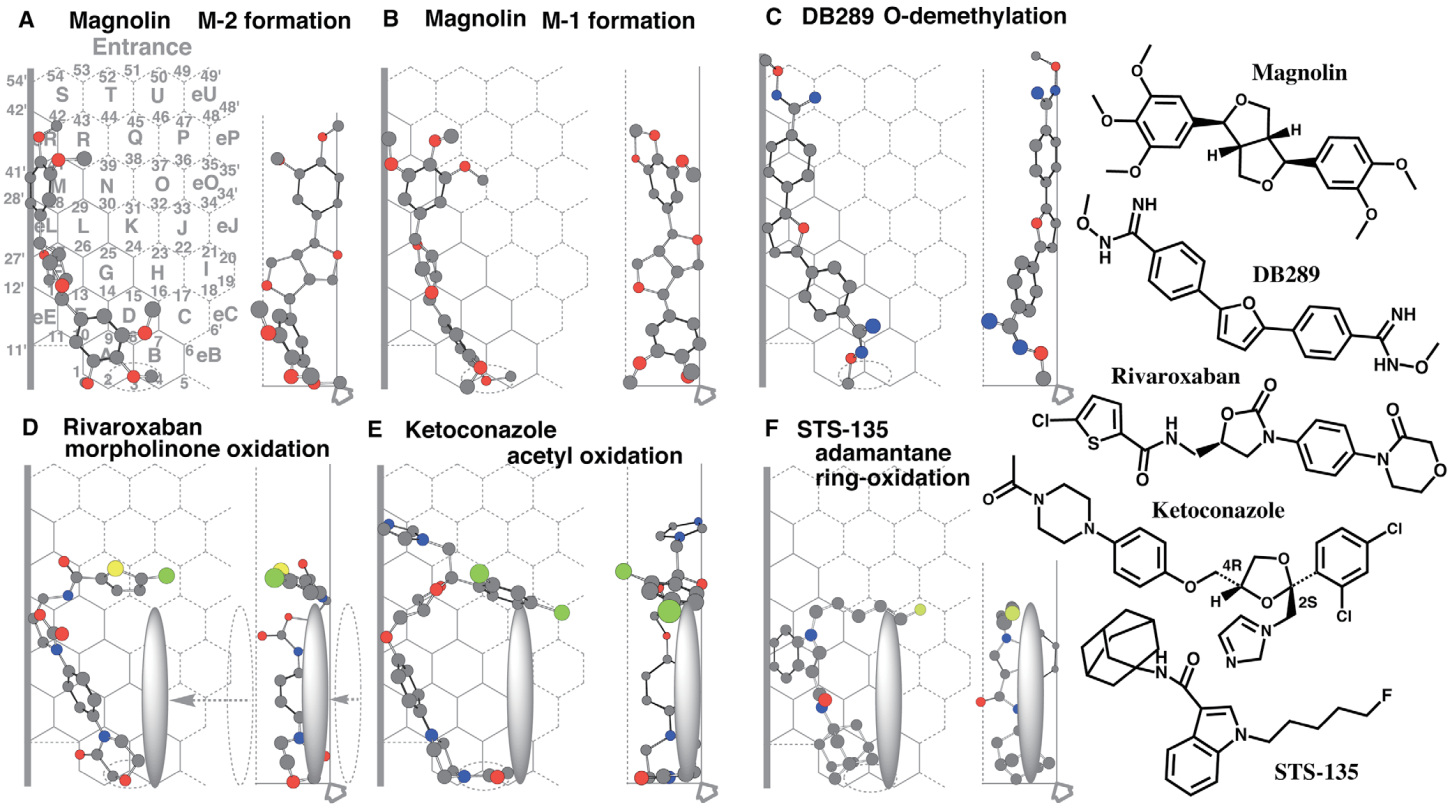
Interactions of slender shape ligands with Left-end and Shelf Placements of magnolin M-2 (A) and M-1 formations (B), of DB289 *O*-demethylation (C), of rivaroxaban morpholinone ring-oxidation (D), of ketoconazole acetyl-oxidation (E), of STS-135 adamantane ring-oxidation (F) are shown as cylindrical shapes of 3D structures. Ligands are also indicated as 2D structures on the right. The movement of Trigger-residue from the initial position (dotted pillar symbol) to the ligand contact (gray pillar symbol) is shown in D.

Placements of magnolin for the M-2 and M-1 formations were generated at Rings B-A(D)-E-F-eL-M-eR(R) (Fig. 4A) and at Rings B-A-E-F-L-M(eR)-R-N (Fig. 4B), respectively. The 3.4-dimethoxyphenyl part contacted with both Left-end and Rear-wall and also contacted with Shelf (Fig. 4A). These contacts stabilized the sitting of the magnolin molecule for M-2 formation.

Contacts with Left-end and Facial-wall of the magnolin molecule might not be tight enough for the M-1 formation as compared to the M-2 formation. These differences might be reflected in their oxidation rates.

DB289 (pafuramidine) undergoes CYP2J2-mediated *O*-demethylation of the *N*-methoxyacetimidamide part^37^^)^.

A placement of DB289 was available for the *O*-demethylation at Rings A-D-E-G-F-eL(L)-M-eR(R)-S (Fig. 4C). Another placement for the *O*-demethylation was constructed at Rings A(B)-E-F(G)-eL(L)-M(N)-R-T(S)-U (Data not shown) in a way the furan part to contact with Left-end. In the latter placement, the Rear-wall contact with both the terminal *N*-methoxyacetimidamide parts was unlikely to be stable enough to support the sitting.

Placements of magnolin and DB289 (Figs. 4A and C) suggested the standing of Left-end at least until the left side of Ring S of Template.

### 3.5 Upper Part of the Bent Ligand Molecules

Standing of Left-end until Ring S (Position 54’) suggested the turning of flexible ligand molecules after Left-end contacts. Sittings of the upper parts of the bent ligands were thus examined on Template.

CYP2J2 mediates the oxidation of the morpholinone part of rivaroxaban^38^^)^.

A placement of rivaroxaban was generated for the morpholinone oxidation at Rings A(B)-E(G)-F-eL(L)-M-R-N-O (Fig. 4D). The molecule contacted Left-end with the oxazolidine-2-one part and then the terminal part was turned in the right direction on CYP2J2-Template. The terminal 2-chlorothiophene ring sat at the middle of Width-gauge at Rings N and O. The region was just above the expected Trigger-residue approaching Position.

Ketoconazole is expected to undergo CYP2J2-mediated oxidation of the terminal *N*-acetyl part^10^^,^^39^^)^. A placement for the terminal oxidation was available at Rings A(B)-E-F-eL-L(M)-N(R-eR)-O-J-eJ (Fig. 4E). The molecule hit Left-end with the ether part and turned in the right direction. The 2,4-dichlorophenyl part contacted with both Facial- and Rear-walls at Ring O, and the imidazole part contacted with both Left-end and Rear-wall at eR region. One of the nitrogen atoms of the piperazine contacted with Shelf, and the other might contribute to the inhibitory action at Ring A.

CYP2J2 mediates mono hydroxylation of the adamantane ring of STS-135 (M-25 formation), although unknown for the exact position^40^^)^.

A placement of STS-135 was generated for the M-25 formation at Rings A(B)-E-F-L(eL)-K-J (Fig. 4F). The indole part contact with both Rear-wall and Left-end. The adamantane part contacted with Shelf and also with Rear-wall to be oxidized. The fluorinated side chain stayed facial-side at Rings K and J. The terminal parts were located just above the expected Trigger-residue approaching Position.

The regional relationship between Trigger-residue approaching Position and terminal parts of bent ligands around Rings O and/or J suggested an additional role of Trigger-residue for supporting the sitting of the bent part of CYP2J2 ligand molecules.

Dronedarone undergoes CYP2J2-mediated ω-oxidations^41^^)^. Placements of dronedarone were generated for the ω-oxidation 1 (presumed dibutylamine part) at Rings A(B)-E(G-H)-F-eL-M-N(R/K-J)-Q-T plus space above Ring T (Data not shown), and for the ω-oxidation 2 (presumed butyl part) at Rings B(A)-D-E(F-eL-M-N)-G(D)-K-O-Q-T-S plus space above Ring T (Data not shown).

CYP2J2 mediates the oxidation of the isopropyl part of ritonavir^42^^)^. A placement was available for the oxidation of the isopropyl part at Rings B-A-E(D)-G(F)-L-M-R(N/S)-Q(O-eO)-U(P)-eU plus a space above eU region (Data not shown). Terminal parts of their bent portions of dronedarone and ritonavir were also located just above the expected Trigger-residue approaching Position. These results supported an idea of the dual role of Trigger-residue, for fastening ligands near Site of oxidation and for supporting the stable standing of bent portions of ligands. Therefore, a presumed Trigger-residue was arbitrarily located as a gray oval of 0.5 x 0.5 x 3 Ring-size dimensions (diameters of horizontal, vertical, and height, Figs. 4D-F).

Trigger-residue is thus expected to behave as follows. Trigger-residue, located initially behind Rear-wall at the right-end of Template, appears between the Facial- and Rear-walls after ligand-descending on Template. Trigger-residue moves to the left and facial directions and ceases after the ligand contact (Fig. 4D). Trigger-residue position in Width-gauge thus may vary depending on the interacting points with ligands.

In addition, the sittings of dronedarone and ritonavir above Ring T and eU region suggested ligand entering from the top of CYP2J2-Template. The Template was, however, not attempted to expand due to the sitting of flexible parts of ligands in this area, Entrance was rather set at the line connecting Position 49’ and Position 54’on Template (Fig. 4A).

### 3.6 Placements of Diagnostic Substrates of CYP2J2

Astemizole undergoes CYP2J2-mediated *O*-demethylation^4^^,^^5^^,^^43^^)^.

**Fig. 5.**
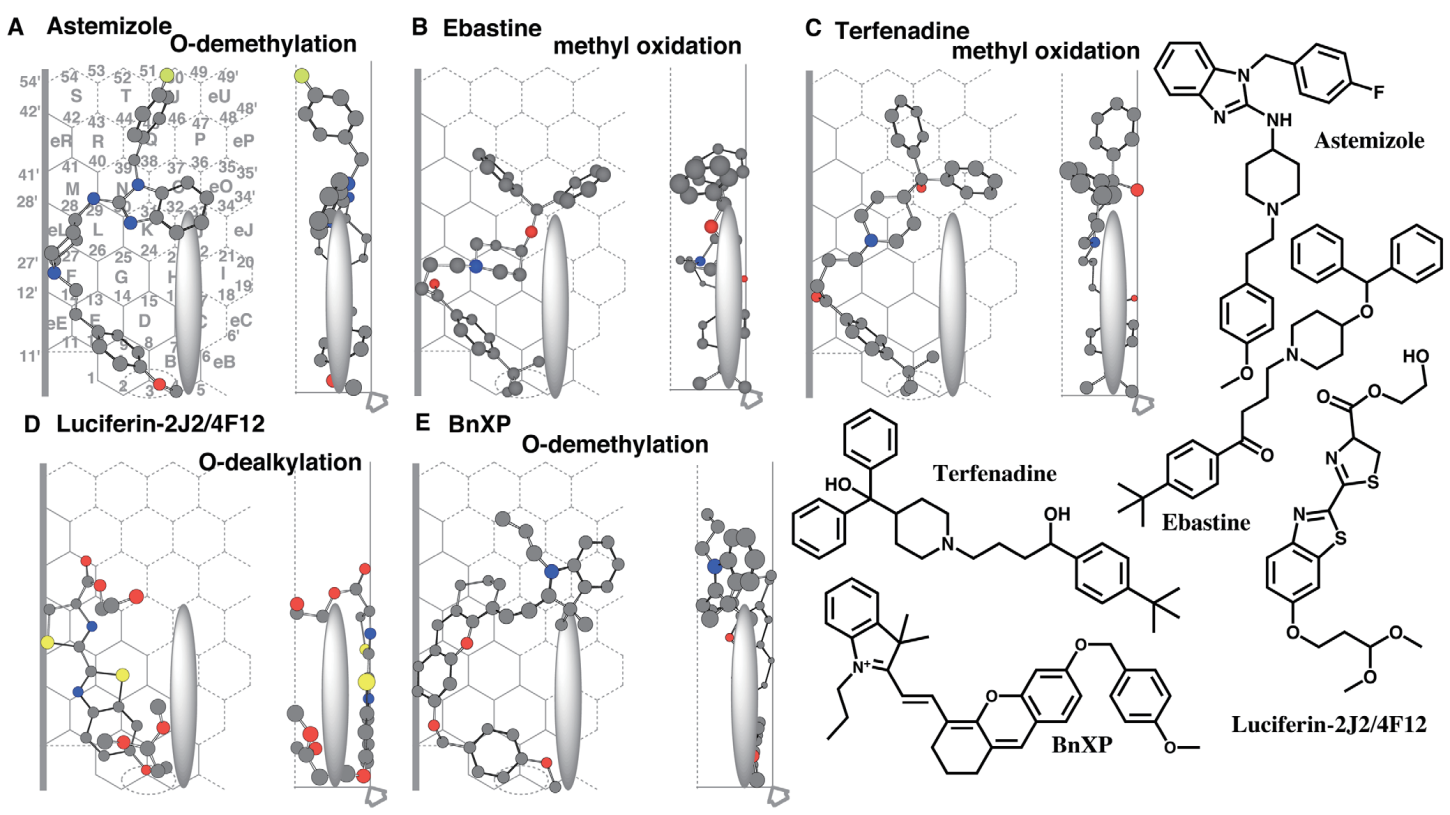
Placements of diagnostic substrates of CYP2J2 and pillar-shape Trigger-residue Placements of astemizole *O*-demethylation (A), of ebastine methyl-oxidation (B), of terfenadine methyl-oxidation (C), of luciferin-2j2/4F12 *O*-dealkylation (D), and of BnXP *O-*demethylation (E) are shown as cylindrical shapes of 3D structures. Ligands are also indicated as 2D structures on the right. Thick arrow at the bottom of Width-gauge indicates the access direction of heme-oxygen atom.

A placement of astemizole was available for the *O*-demethylation at Rings A(B)-E-F-eL-M-N(Q-U)-K-O(J)-eO (Fig. 5A). The sitting of the lower part of the molecule was stabilized by the contact with Rear-wall, Shelf, and Site of oxidation. The upper part contacted Left-end with the piperidine part and turned in the right direction. The fluorophenyl and benzimidazole parts stayed around Rings N(Q-U)-K-O(J) with the Facial-wall contact.

CYP2J2 mediates the oxidation of *tert*-butyl part of ebastine^5^^,^^44^^)^.

A placement of ebastine was generated for the *tert*-butyl oxidation at Rings A(B)-E-F-G(L)-K(N-R)-O-eO (Fig. 5B). The sitting of the *tert*-butylphenylethanone part was stabilized through the contact with Shelf, Left-end and Rear-wall. The upper part was contacted with Facial- and Rear-walls.

CYP2J2 mediates the methyl oxidation of the *tert*-butyl part of terfenadine^6^^,^^45^^)^.

A placement of terfenadine was generated for the methyl oxidation at Rings A(B)-E-F-L(K)-N-O(Q-T)-eO (Fig. 5C). The upper part of terfenadine molecule was stabilized with Facial- and Rear-wall contacts.

Luciferin-2J2/4F12 is a substrate of CYP2J2 and is converted to a chemiluminescent product after the cleavage of the ethereal linkage^46^^)^.

A placement of luciferin-2J2/4F12 was generated for the cleavage of the ethereal linkage at Rings D-B-A-E(G)-F-eL-L-M-N (Fig. 5D). This molecule satisfied contacts with Shelf, Left-end, and Site of oxidation in addition to the contact with Facial and Rear-walls.

CYP2J2 mediates the *O*-demethylation of BnXP to yield a pro-fluorogenic metabolite^47^^)^.

A placement for the *O*-demethylation of BnXP was constructed at Rings A(B)-E(eE)-F-L(eL)-N(M)-K-O(J/Q)-eO(P-eP) (Fig. 5E). This xanthene part of this molecule hit Left-end and contacted with Rear-wall. The contact of the methoxybenzyl part with the Shelf supported the stable sitting around the Site of oxidation. The initial *O*-demethylation and subsequent oxidation to the quinone methide might lead to the generation of a fluorogenic product. The upper part of the molecule contacted Facial- and Rear-walls around Rings eO-O-Q.

As described above in the Section 3.3. (Trigger-residue and the interaction with ligands), Trigger-residue appeared, after the descending of ligands, at an outside space of Ring B and then migrated to the left direction to contact with ligands. Trigger-residue was expected to migrate through a rear side path maximally until Ring B, but not to enter Ring A. The mode of Trigger-residue interaction was consistently applicable for bent ligands of CYP2J2 (Fig. 4D-F and Fig. 5A-E).

These results further supported the additional role of Trigger-residue for the sitting supports of the upper part of CYP2J2 ligands.

A pillar shape of Trigger-residue was thus assumed to appear from the rear side of CYP2J2-Template after the ligand arrival and then contacted with a ligand at both Site of oxidation and the area of Rings K and J (Figs. 4D-F and 5A-E). Of course, further studies are necessary to substantiate the role of Trigger-residue for the sitting supports of the upper part of CYP2J2 ligands. The interacting mode of Trigger-residue was acceptable for all the test ligands on CYP2J2-Template.

### 3.7 Placements of Endobiotics and Bi-molecule Binding

CYP2J2 mediates the 25-oxidation of vitamin D_3_ and 25-hydroxyvitamin D_3_^48^^)^.

**Fig. 6.**
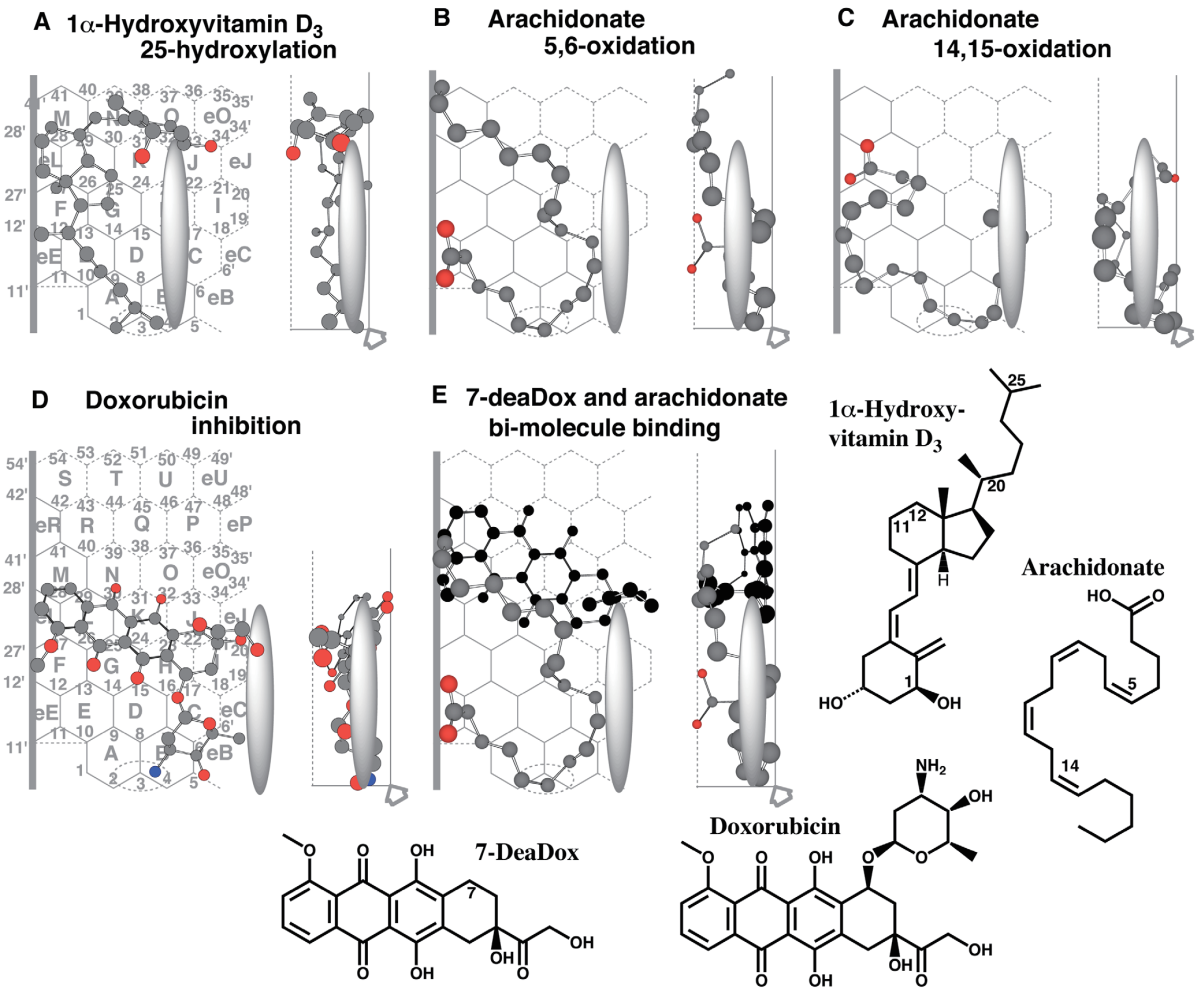
Placements of endobiotics Placements of 1α-hydroxyvitamin D_3_ 25-oxidation (A), of arachidonate 5,6- (B) and 14,15-oxidations (C), of doxorubicin inhibition (D), of bi-molecule binding of 7-deaDox and arachidonate for the 5,6-oxidation (E) are shown as cylindrical shapes of 3D structures. Trigger molecule (7-deaDox) is shown in dark color. Ligands are also indicated as 2D structures with parts of their position numbers. Thick arrow at the bottom of Width-gauge indicates the access direction of heme-oxygen atom.

A placement of 1-hydroxyvitamin D_3_ was available for the 25-oxidation at Rings A(B)-E-F(G)-L(eL)-N-O(K)-J (Fig. 6A). Both the C_11-12_ at ring C and methyl part at C_20_ contacted with Left-end. This molecule was expected to contact Trigger-residue at both the terminal parts around Ring B and Ring J. Vitamin D_3_ and D_2_ took placements for their 25-oxidations similar to 1-hydroxyvitamin D_3_ (Data not shown).

CYP2J2-mediates the epoxidation of arachidonic acid at the 5,6-, 8,9-, 11,12- and 14,15-positions^1^^,^^49^^)^.

Placements of arachidonic acid were generated for their 5,6- and 14,15-oxidations at Rings eE-E-A-B-C-H-K-L-M-eR (Fig. 6B) and Rings eL-L-G-F-eE-E-A-B-C-H (Fig. 6C), respectively. The former arachidonate molecule contacted Left-end with both terminal parts, whereas the latter arachidonate contacted Left-end with the middle part. Both the molecules sat on Shelf.

Doxorubicin (adriamycin) inhibits CYP2J2-mediated oxidation of arachidonate^49^^)^.

A placement of doxorubicin was generated for the inhibition at Rings eB-B-C-I-H(D/K)-G-L-(F)-eL (Fig. 6D). The amino group on the sugar part would interact with heme.

A de-glycosylated derivative, 7-deaDox, modulates CYP2J2-mediated oxidations of arachidonate to increase the rate of 5,6-oxidation but to decrease the rate of 14,15-oxidation^49^^)^.

A placement of arachidonate for the 5,6-oxidation was constructed as bi-molecule binding at Rings eO-O-Q(N)-R(M/T)-S(eR) for 7-deaDox (Fig. 6E). The upper part of arachidonate molecule sat at facial side for the 5,6-oxidation. Most of the 7-deaDox molecule stayed rear side space, and the methoxy part around Left-end (eL region) held the arachidonate molecule to enhance the sitting stability of the arachidonate molecule.

The carboxylic aid terminal was located at rear side space for the 14,15-oxidation (Fig. 6C). The placement of 7-deaDox molecule was expected to compete with the arachidonate molecule for the 14,15-oxidation, and thus to reduce the chance of the 14,15-oxidation. These results were consistent with the experimental data on the influence of 7-deaDox toward CYP2J2-mediated arachidonate oxidations.

## 4 Discussion

The present study has established a fused hexagonal-grid Template system for CYP2J2, as a tool to understand metabolisms of the ligands. Over 110 reactions of 92 distinct chemicals published as CYP2J2 ligands have been examined in the Template system. CYP2J2 ligands are assumed to enter from the right top of the Template (Entrance). CYP2J2-Template has Facial-wall and Rear-wall separated parallelly by 1.5 Ring size of the width, which has been determined empirically through ligand assembly in the present study.

CYP2J2 ligands start to contact Rear-wall simultaneously with the plural sites. The ligands then hit with Facial-wall and Left-end, and often sat on Shelf. Parts of the ligands located at Site of oxidation are fastened with the aid of Trigger-residue and subjected to catalyses. Trigger-residue appears through Rear-wall. The residue moves from a space around eB region to a Position near the border of Rings A and B, if allowed to migrate. CYP2J2 ligands thus need to sit on Ring B to be metabolized (Section 3.2 Minimal occupancy at the bottom region**)**. A similar limitation of Trigger-residue movement is also observed with CYP2C9- and CYP2C19-Template systems^19^^,^^21^^)^. All the good ligands of CYP2J2 examined are fixed with Left-end and Trigger-residue on the Template.

Often CYP2J2 ligands turned in the right direction after the Left-end contact. The upper portions are stabilized with Facial- and/or Rear-wall contacts, and possibly also the contact with a pillar shape of Trigger-residue.

Trigger-residue, introduced into the system for ligand fastening and initiation of catalysis, was thus expected to contribute to ligand holding at dual sites on CYP2J2-Template.

In the current study, cyclosporin A was unable to be accommodated within CYP2J2-Template. The molecule for M1 formation^28^^)^ occupies an area outside of Rings eJ, eO and eP and is not included completely within Width-gauge (Data not shown). Although the reason for the accommodation failure is unclear, this area may be used also for ligand entrance and have an extended width to accept plump structures of CYP2J2 ligands.

Most of CYP2J2 ligands are expected to interact as uni-molecule binding on Template. A few phenomena are explained as bi-molecule binding of non-identical ligands (Section 3.7 Placements of endobiotics and bi-molecule binding). Similar to bi-molecule bindings of other P450 enzymes, a substrate that sat at Site of oxidation was termed “pro-metabolized molecule” and the second substrate was termed “trigger molecule” on CYP2J2 Template.

Pro-metabolized and trigger molecules need to have a slight overlapping point(s) on Template, although trigger molecules are situated behind, without direct contacts, to contribute to the immobilization of pro-metabolized molecules. Selective enhancement of arachidonate 5,6-oxidation, but not the 14,15-oxidation, with 7-deaDox (Fig. 6E) was consistent with the experimental results in the recombinant CYP2J2 system.

In the present study, CYP2J2 has been characterized to share the mode of ligand interaction with CYP2B6, CYP2C8, CYP2C9, CYP2C18, and CYP2C19. These P450 enzymes have common components such as Left-end, Shelf, and Facial- and Rear-wall standing parallelly on Templates. In addition, ligand interactions are mutually expected to start with Simultaneous plural-contact with Rear-wall in these Template systems. CYP2J2 and CYP2Cs have equivalent sizes (1.5 Ring size) of the distance between Facial- and Rear-walls (Width-gauge), but was less than the Width-gauge of CYP2B6 (1.7 Ring size).

Fused grid-based Template systems of human CYP enzymes have been applied for studies of chemical metabolisms and safety evaluations of natural and industrial substances due to the advantages of the prediction accuracy and deciphering properties for possible causes of observed regioselectivity and poor metabolisms,^15^^,^^16^^,^^25^^,^^50^^,^^51^^)^. The refined CYP2J2-Template system will further strengthen the broad applicability of the fused grid-based Template systems. After the completion of the present study, CYP2J2 genotype was reported to associate with the metabolism of praziquantel in patients of *S. mansoni* infection^52^^)^. Placements of *R*- and *S*-praziquantel were generated for their 4’-oxidations at both Rings A(B)-E-G(H)-K-L(eL)-M (Data not shown) on CYP2J2-Template.

## Terms Used for Template System

**Entrance:** A space at the top of Template for ligand entering

**Facial-wall and Rear-wall:** Vertically standing parallel walls at the facial and rear borders of Template

**Left-end**: The left-side border of Template located between Entrance and Shelf

**Pro-metabolized molecule:** Substrates to be oxidized or reduced are termed as “pro-metabolized molecule” in the simulation experiment.

**Shelf:** A plateau-like shape area between Position 10 and Left-end of CYP2J2 Template (Position 11’)

**Simultaneous plural-contact:** Initial interactions of CYP2J2 ligands start with their simultaneous contact with plural-points to Rear-wall standing upright at rear end of Template.

**Site of oxidation:** A confined space for enzymatic oxidation(reduction). An area between Positions 2 and 4 corresponds to Site of oxidation in CYP2J2 Template

**Trigger molecule:** A molecule, which is not oxidized, acts for triggering the catalysis in bi-molecule binding. Trigger molecules need to have direct contacts to pro-metabolized molecules on 2D Template

**Trigger-residue movement:** Trigger-residue was located originally around eB region and appeared from Rear-wall to move to the left direction to fasten ligand molecules.

**Width-gauge:** A guide tool to judge allowable width for ligand accommodation around Template which was determined empirically as 1.5 Ring-size of thickness for human CYP2J2.
